# Severe Pancytopenia Secondary to Combined Vitamin B12 and Folate Deficiency Mimicking Bone Marrow Failure: A Case Report

**DOI:** 10.7759/cureus.98385

**Published:** 2025-12-03

**Authors:** Ali Sheikh, Lally De Soysa, Fath Mohammed

**Affiliations:** 1 Emergency Medicine, Wrexham Maelor Hospital, Wrexham, GBR; 2 Haematology, Wrexham Maelor Hospital, Wrexham, GBR

**Keywords:** b12, folate deficiency, folic acid, severe pancytopenia, vit b12 deficiency

## Abstract

Vitamin B12 and folate are essential cofactors for DNA synthesis and red cell maturation. Severe combined deficiency is rare but can mimic bone marrow failure by causing profound pancytopenia and intramedullary haemolysis. This report describes the case of a 65-year-old woman who presented with profound pancytopenia and jaundice caused by severe combined vitamin B12 and folate deficiency. The identification of megaloblastic changes and prompt administration of vitamin replacement led to complete recovery. This case underscores the importance of considering nutritional deficiencies when diagnosing pancytopenia. The report highlights the continued prevalence of such deficiencies among older adults in the United Kingdom and the subsequent associated morbidity.

## Introduction

Vitamin B12 and folate deficiencies are common causes of megaloblastic anaemia, but can occasionally present with severe pancytopenia. Pancytopenia is defined as the reduction in all three major cell lines, with haemoglobin levels falling below 120 g/L in women, the white blood cell count dropping below 4 × 10⁹/L, and platelet levels falling below 150 × 10⁹/L. The causes of pancytopenia can be grouped into four broad mechanisms: decreased bone-marrow production, increased peripheral destruction or consumption, sequestration within the reticuloendothelial system, and congenital disorders [1].

Vitamins are vital cofactors in DNA synthesis and one-carbon metabolism. Deficiency of either hampers thymidylate synthase activity, resulting in faulty deoxythymidine triphosphate production, nuclear cytoplasmic asynchrony, and ineffective haematopoiesis [2-4]. In the United Kingdom, national surveys estimate that 5-10% of adults aged 65 years and over have biochemical B12 deficiency, rising to nearly 20% in those aged 75 years and over; 8-12% show folate deficiency, with a further 20% in marginal status [5,6]. Polypharmacy, malnutrition, and the use of proton pump inhibitors (PPIs) or metformin contribute significantly.

We report a case of combined B12 and folate deficiency presenting as profound pancytopenia in an elderly woman, demonstrating how nutritional causes can mimic primary marrow failure and highlighting the importance of early diagnosis of malnutrition. This case highlights the importance of considering nutritional deficiencies in the differential diagnosis of pancytopenia, even during haematological recovery.

## Case presentation

A 65-year-old woman presented with six months of progressive tiredness, loss of appetite, and weight loss, associated with new-onset mild confusion. She denied bleeding, fever, abdominal pain, or bowel disturbance. She reported a chronically poor appetite and typically consumed only half of a microwave meal once or twice daily. Her past medical history included multiple sclerosis, hypertension, osteoarthritis, and hiatus hernia. At the time of admission, she was taking pregabalin 25 mg three times daily for neuropathic pain and had recently stopped omeprazole 20 mg once daily for her hiatus hernia. She lived alone, did not smoke, and abstained from alcohol.

On examination, she appeared markedly pale and jaundiced, without lymphadenopathy or hepatosplenomegaly. There were no signs of glossitis or cheilitis. Cardiovascular and respiratory examinations were unremarkable. Neurological examination revealed mild gait ataxia consistent with her baseline multiple sclerosis. There was no glove-and-stocking peripheral neuropathy, no dorsal column impairment, and no neurological features typically associated with vitamin B12 deficiency.

**Figure 1:**
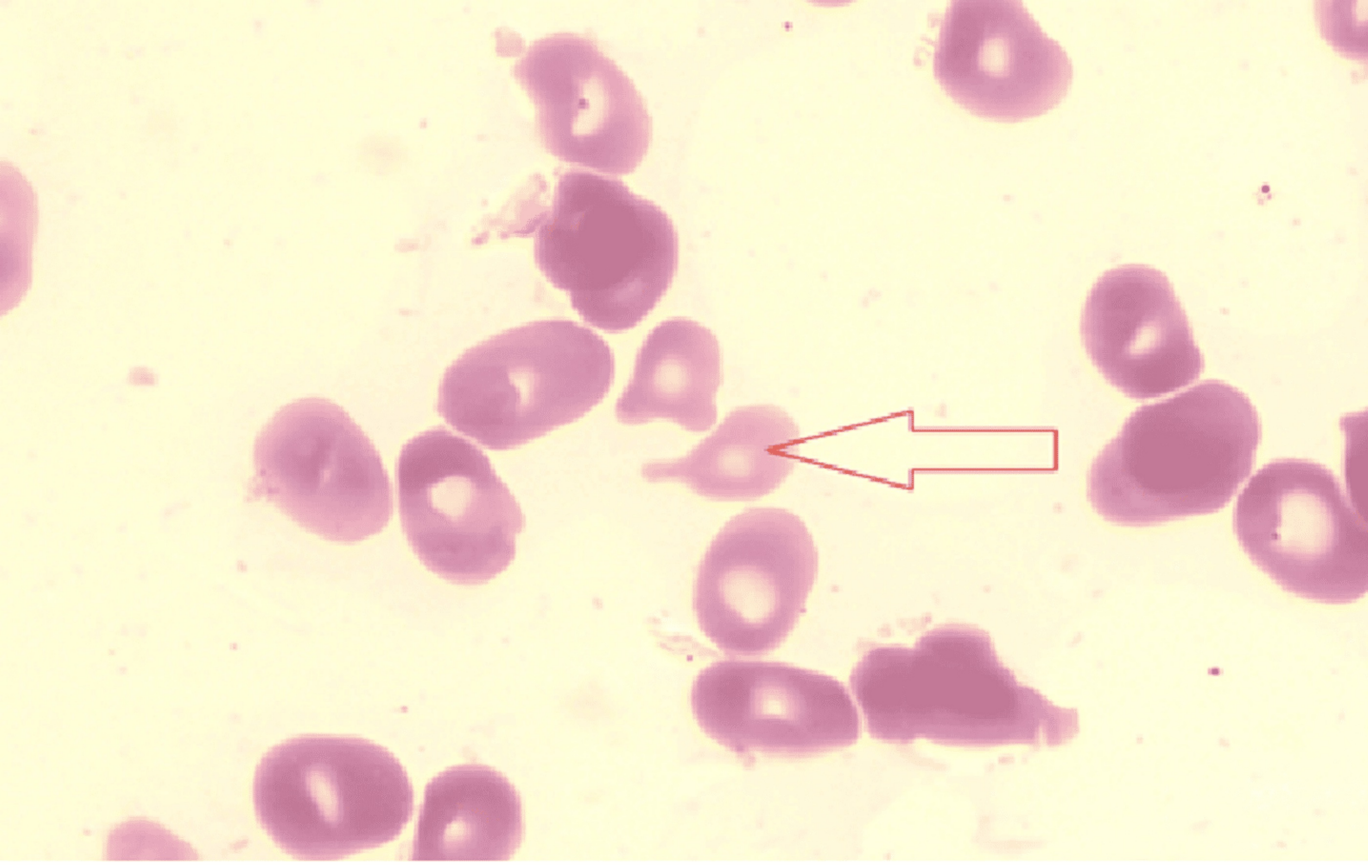
Blood smear shows a tear drop cell (arrow) typical of megloblastic anaemia

**Figure 2:**
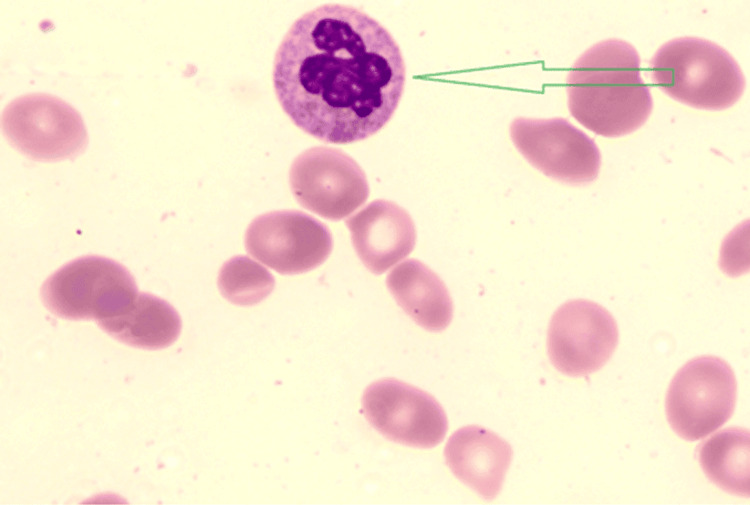
Peripheral blood film showing macro-ovalocytes and hypersegmented neutrophil (arrow), typical of megaloblastic anaemia.

Initial investigations revealed profound pancytopenia: haemoglobin 30 g/L, white cell count 0.6 × 10⁹/L, platelets 18 × 10⁹/L, and mean corpuscular volume (MCV) 122 fL. The peripheral blood film demonstrated macro-ovalocytes, hypersegmented neutrophils, and occasional tear-drop and pencil cells, typical of megaloblastic anaemia (Figures 1, 2).

Serum bilirubin 147 µmol/L and lactate dehydrogenase (LDH) > 1800 U/L indicated intramedullary haemolysis, while haptoglobin was undetectable. Vitamin B12 114 ng/L (180-900) and folate 1.4 µg/L (>3.0) confirmed combined deficiency; anti-intrinsic factor antibodies were negative. Methylmalonic acid and homocysteine assays were not available at our centre; however, the diagnosis of combined vitamin B12 and folate deficiency was supported by the biochemical levels, characteristic blood-film morphology, and the patient’s clear haematological improvement following replacement therapy. Key laboratory values with reference ranges are summarised in Table 1.

**Table 1:** Serial haematological and biochemical parameters showing recovery following B12 and folate replacement “–” = not done. MCV: mean corpuscular volume; CRP: C-reactive protein; LDH: lactate dehydrogenase

Investigation	Reference Range (Units)	Day 1	Day 2	Day 3	Day 4	Day 5	Day 6	Day 10	Day 12	Day 17	Day 25	Day 30
WBC	4.0–11.0 ×10⁹/L	0.6	0.8	1.2	1.5	1.9	6.3	11.7	7.9	4.3	3.2	4.1
Hemoglobin	115–165 g/L	30	48	71	81	76	89	91	112	96	89	101
Platelets	150–400 ×10⁹/L	30	18	29	14	13	21	144	223	209	109	152
RBC	3.8–5.5 ×10¹²/L	1.34	0.68	2.14	2.56	2.43	2.78	2.9	3.41	2.96	2.6	3.0
MCV	80–100 fL	102	122	96	94	93	96	101	103	105	105	103
Neutrophils	1.7–7.5 ×10⁹/L	0.3	0.3	0.3	0.2	0.3	6.3	9.2	5.5	3.0	0.3	0.4
Monocytes	0.2–0.8 ×10⁹/L	0	0	0	0.4	0.6	1.99	1.4	1.7	0.4	3.0	1.0
CRP	< 5 mg/L	12	6	–	–	–	2	< 1	3	4	17	15
Bilirubin	< 21 µmol/L	108	147	121	73	55	61	34	–	–	186	–
LDH	< 250 U/L	> 1800	> 1800	> 1800	–	1665	–	632	–	379	–	–
Folate	> 3.0 µg/L	–	1.4	–	–	–	–	–	–	> 20	–	–
Vitamin B12	180–900 ng/L	114	116	–	–	–	–	–	–	> 2000	–	–
Anti-Intrinsic-Factor Ab	< 20 U/mL	–	< 0.3	–	–	–	–	–	–	–	–	–
Haptoglobin	0.3–2.0 g/L	–	< 0.1	–	–	–	–	–	–	–	–	–
Reticulocyte count	20–80 ×10⁹/L	–	19	20	21	47	225	–	–	–	69	64

Because of the mild confusion on presentation, a CT head (Day 2) was performed and showed no evidence of acute intracranial haemorrhage, surface collection, or skull fracture. In view of the raised bilirubin, a CT abdomen-pelvis (CTAP) was also undertaken to exclude biliary or hepatic pathology. This demonstrated no features of complicating cholecystitis or biliary dilatation, with low-volume ascites around the liver and a small right pleural effusion with scattered ground-glass changes at the lung bases. There were no radiological features suggestive of intra-abdominal or haematological malignancy.

**Figure 3:**
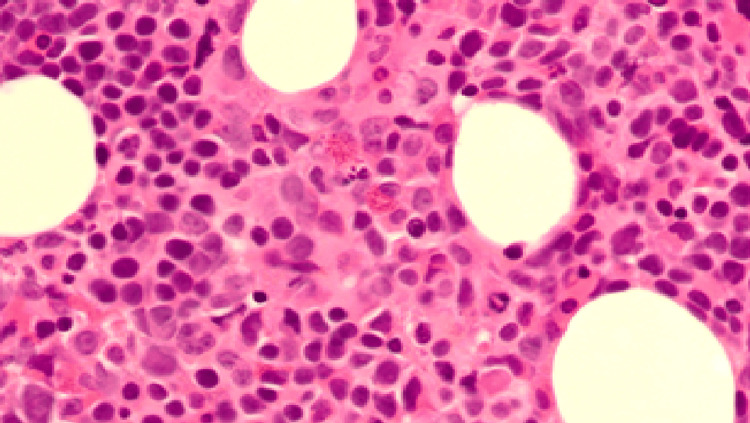
Bone marrow trephine (low power) shows evidence of dyserythropoiesis and myeloid maturation arrest, with megakaryocytes of variable maturity, consistent with megaloblastic changes.

**Figure 4:**
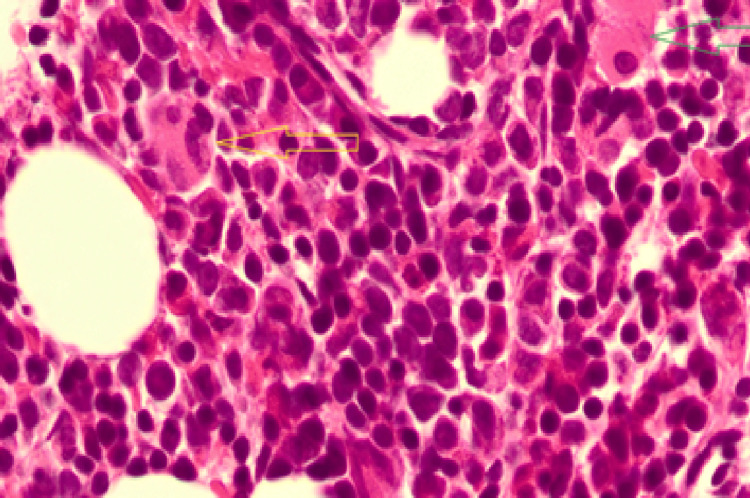
Bone marrow trephine (high power) showing megaloblasts with nuclear cytoplasmic asynchrony (arrows), consistent with megaloblastic change due to vitamin B12 and folate deficiency.

Bone marrow biopsy revealed a hypercellular marrow with dyserythropoiesis and myeloid maturation arrest, confirming megaloblastic change (Figures 3, 4). Imaging excluded any infiltrative or malignant cause of marrow suppression.

The patient received six units of packed red cells and one unit of platelets during the first three days of admission. Parenteral hydroxocobalamin 1 mg intramuscularly on alternate days and oral folic acid 5 mg daily were commenced immediately. Filgrastim for two days and prednisolone 20 mg daily for six days were used to support marrow recovery.

Within 10 days, haemoglobin increased to 91 g/L, platelets to 223 × 10⁹/L, and white cell count to 11.7 × 10⁹/L, confirming reversible bone-marrow failure secondary to combined vitamin B12 and folate deficiency. Blood counts remained stable and within near-normal ranges on Day 25 and Day 30, reinforcing the complete reversibility of this nutritional marrow failure once appropriate vitamin therapy was initiated. Furthermore, the rapid normalisation of counts after vitamin therapy provided direct evidence that the bone-marrow failure was fully reversible once the nutritional deficiencies were corrected.

Subsequent iron studies summarised in Table 2 showed iron 6 µmol/L, transferrin 1.4 g/L, and saturation 17%, reflecting increased erythropoietic demand during recovery; intravenous iron corrected this. She was discharged after nutritional optimisation and remains well on follow-up.

**Table 2:** Iron studies which explain the slight decrease in hemoglobin on day 25 compared to day 19

Investigation	Reference Range (Units)	Result (Day 25)
Serum Iron	10–30 µmol/L	6 µmol/L
Transferrin	2–4 g/L	1.4 g/L
Transferrin Saturation	20–50 %	17.10%

## Discussion

This case demonstrates a reversible cause of apparent bone marrow failure due to combined vitamin B12 and folate deficiency. The patient presented with profound pancytopenia and biochemical signs of intramedullary haemolysis, significantly raised LDH, indirect hyperbilirubinaemia, and undetectable haptoglobin, consistent with ineffective haematopoiesis [2,3,7,8].

Pathophysiology

Vitamin B12 and folate are essential in one-carbon metabolism and in synthesising thymidylate and purines from scratch. A deficiency in either vitamin can impair thymidylate synthase activity, leading to problems with DNA replication, a mismatch between the nucleus and cytoplasm, and premature death of erythroid, myeloid, and megakaryocytic precursors in the bone marrow [2-4,7,9]. Consequently, levels of LDH and indirect bilirubin rise, while the reticulocyte count drops, indicating intramedullary rather than peripheral haemolysis [10]. When folate deficiency coexists, haematological failure becomes more severe. Folate donates methyl groups for thymidylate synthesis, while vitamin B12 regenerates tetrahydrofolate (THF) from 5-methyl-THF. Deficiency of either compound blocks DNA synthesis at multiple stages, leading to a near-complete arrest of erythropoiesis. In B12 deficiency, folate becomes “trapped” as 5-methyl-THF (the methyl-folate trap), producing a functional folate deficiency even when serum levels are normal [11]. B12 deficiency usually develops gradually because the liver stores 2-5 mg of it. However, this patient’s haemoglobin dropped sharply from 161 g/L to 30 g/L within just six months, indicating rapid failure of bone marrow function. Factors contributing included reduced food intake, long-term use of omeprazole, and coexistent folate deficiency, all impairing cobalamin absorption and accelerating marrow exhaustion [2-4,7,11,12].

Diagnostic considerations

The patient was prescribed 5 mg of folic acid daily for two months following a general practitioner (GP) visit three months before admission, due to reduced appetite and a folate level of 1.1. At that time, no B12 or haemoglobin testing was performed. A full blood count might have revealed macrocytic anaemia. Folate therapy can temporarily relieve macrocytosis but may mask or worsen B12 deficiency by driving residual DNA synthesis without resolving the methylation block, potentially increasing neurological risk and precipitating pancytopenia once hepatic stores are exhausted [1,11].

Management and outcomes

In this patient, urgent treatment was essential. A combination of red blood cells, platelets, and parenteral hydroxocobalamin (1 mg IM every other day) plus oral folic acid (5 mg daily) was initiated immediately. A rapid reticulocyte response occurred within five days and nearly complete recovery by two weeks, confirming marrow reversibility. As per guidelines, oral maintenance therapy followed parenteral repletion. Vitamin B12 should always be given before or with folate to prevent neurological injury. Vitamin B12 deficiency affects 3-5% of the general population and up to 20% of those aged 75 and over [2,12]; folate deficiency remains common due to poor intake, malabsorption, and medication effects. Both deficiencies are associated with frailty and cognitive decline [2,4,12]. This case highlights that even in developed healthcare systems, nutritional deficiencies remain important but reversible causes of pancytopenia. Early recognition and prompt supplementation can prevent unnecessary invasive investigations, reduce morbidity, and improve outcomes [13]. The patient's haemoglobin decreased to 89 on day 25, and iron studies indicated iron deficiency, likely reflecting increased iron demand during recovery. As vitamin B12 and folate levels were replenished, erythropoiesis accelerated, depleting iron stores. This was corrected with IV iron infusion, after which Hb rose to 101g/dL.

Learning points

There were several lessons learnt from this case: (i) Combined vitamin B12 and folate deficiency can lead to pancytopenia, resembling bone marrow failure, (ii) Macrocytosis with elevated LDH and indirect bilirubin indicates intramedullary haemolysis, (iii) Folate should never be given on its own if B12 deficiency could be present, (iv) Parenteral hydroxocobalamin effectively addresses deficiencies in symptomatic or malabsorptive conditions, (v) In older adults, include a full blood count, B12, and folate in the assessment of appetite loss or weight change, especially with long-term PPI use, and (vi) Iron deficiency can develop after folate and/or B12 supplementation, probably because of increased cellular demand.

## Conclusions

This case demonstrates that a rare combined deficiency of vitamin B12 and folate can manifest as severe pancytopenia, mimicking bone marrow failure. Recognising megaloblastic changes and administering vitamin supplements promptly resulted in full recovery, avoiding additional complications. This case also highlights that, even when diagnosis is delayed, prompt supplementation once the nutritional deficiency is recognised can lead to full haematological recovery.
